# A Case Report of *Bordetella holmesii* Concomitant Vascular Graft Infection and Native Vertebral Osteomyelitis

**DOI:** 10.1155/crdi/5528529

**Published:** 2026-05-19

**Authors:** Joya-Rita Hindy, Ryan W. Stevens, Sergio L. Alvarez-Mulett, Aaron J. Tande, Walter R. Wilson, Aditya S. Shah

**Affiliations:** ^1^ Division of Public Health, Infectious Diseases and Occupational Medicine, Mayo Clinic, Rochester, Minnesota, USA, mayo.edu; ^2^ Department of Pharmacy Services, Mayo Clinic, Rochester, Minnesota, USA, mayo.edu

**Keywords:** *Bordetella holmesii*, case report, native vertebral osteomyelitis, vascular graft infection

## Abstract

*Bordetella holmesii* is a fastidious Gram‐negative coccobacillus that was first isolated in 1983. It is an infrequent human pathogen, predominantly limited to asplenic and other immunocompromised hosts. We describe herein the first reported case of vascular graft infection caused by *B. holmesii* with concomitant native vertebral osteomyelitis in an immunocompetent patient.

## 1. Introduction

*Bordetella holmesii* is a Gram‐negative, coccobacillus within the genus *Bordetella*. It was first isolated in 1983 by microbiologist Barry Holmes from a patient with asplenia and then formally named in 1995 in his honor [1]. Since its identification, *B. holmesii* has been reported as an infrequent cause of invasive infection, including bacteremia, meningitis, pericarditis, pneumonia, septic arthritis of native and prosthetic joints, predominantly in hosts with asplenia, and other immunocompromising conditions. It was also associated with pertussis‐like respiratory symptoms in healthy individuals [2, 3]. Isolation of *Bordetella* spp. can be challenging secondary to its fastidious nature and slow growth pattern [2, 3]. To our knowledge, this is the first reported case of a vascular graft infection (VGI) caused by *B. holmesii* with concomitant discitis/native vertebral osteomyelitis (NVO) in an immunocompetent patient. It also underscores the value of advanced diagnostic tools, such as molecular testing and genomic sequencing, in identifying uncommon bacterial causes of invasive infectious diseases.

## 2. Case Presentation

A 78‐year‐old male with a history of infrarenal abdominal aortic aneurysm status post endovascular repair with aortobiiliac stent graft 6 years ago, prostate adenocarcinoma treated with prostatectomy and radiation therapy over 20 years ago, chronic right‐sided hydronephrosis status post nephrostomy tube placement, hypertension treated with amlodipine, losartan, and torsemide, hyperlipidemia treated with atorvastatin, CKD Stage IIIb, depression treated with fluoxetine, and bilateral inguinal hernia repair presented to a local clinic with 8 weeks of constant lower back pain unrelieved by over‐the‐counter analgesics, without fever or chills. He worked a desk job, and had no relevant exposures such as hunting, fishing, or farming. His clinical frailty scale was 2‐3. He reported alcohol consumption 3 times per week, no smoking tobacco, no animal exposure apart from contact with his domestic cats, and lived with his wife in an urban setting.

**FIGURE 1:**
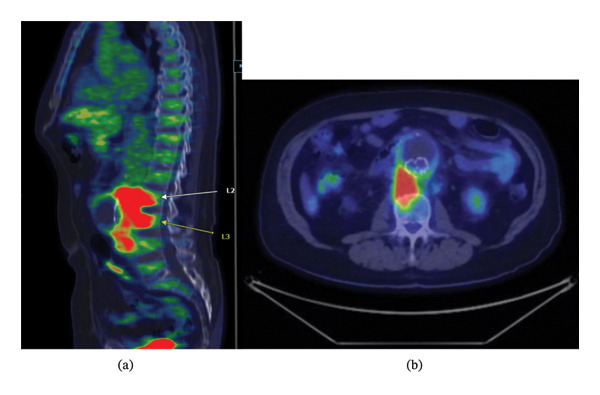
F‐18 FDG PET/CT skull to thigh. Sagittal view (a) and transversal view (b).

He initially had a computed tomography angiogram (CTA) scan of the abdomen and pelvis as part of his aortic graft follow‐up, which showed abdominal lymphadenopathy. A subsequent fluorodeoxyglucose (FDG) positron emission tomography/computed tomography (PET/CT) skull to thigh scan (Figure 1) performed for further evaluation revealed extensive hypermetabolic soft tissue attenuation along the entire course of the aortic stent graft, especially near L2‐L3 vertebrae, consistent with possible stent graft infection. The hypermetabolic uptake also involved the anterior aspect of the L2‐L3 vertebral bodies and surrounded the L2‐L3 disc space, raising concern for associated L2‐L3 NVO. Notably, there was a posterior type 1 endoleak from proximal graft and a possible types 2 and 3 endoleak from the posterior aspect of the right limb of the infrarenal AAA endograft.

**FIGURE 2:**
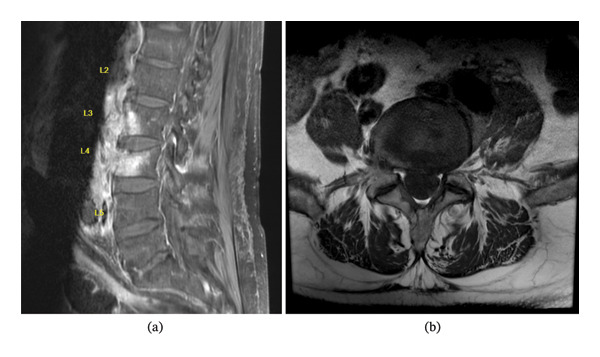
Lumbar spine MRI: sagittal view (a) and axial view (b).

A subsequent lumbar spine magnetic resonance image (MRI) with and without IV contrast (Figure 2) confirmed phlegmonous changes in the midline/right L2‐L3 prevertebral space, adjacent to the abdominal aneurysmal sac and stent graft. Mild thickening and enhancement of the posterior wall of the aneurysmal sac were noted, concerning for an infectious process. There was bone marrow signal alteration in L2‐L3 vertebral bodies, suggestive of NVO, without evidence of an epidural abscess.

The patient was not started on any antimicrobials and was admitted to the hospital for a CT‐guided biopsy of the phlegmon in the midline/right L2‐L3 prevertebral space. The patient was afebrile and hemodynamically stable. Tests obtained from the biopsy sample were unrevealing, including fungal 28S deoxyribonucleic acid (DNA) polymerase chain reaction (PCR), 16S ribosomal ribonucleic acid (rRNA) PCR, mycobacterial culture, aerobic and anaerobic bacterial cultures, fungal culture. Additional blood work revealed a white blood cell (WBC) count of 6.11 (4.00–11.00 10^3^/μL), creatinine 0.98 (0.8–1.3 mg/dL), C‐reactive protein (CRP) of 8.1 (0.0–0.5 mg/dL), erythrocyte sedimentation rate (ESR) 52 (0–15 mm/h), bacterial blood cultures with no growth after 14 days of incubation, and negative *Bartonella* IgM, IgG, and DNA PCR from blood. A plasma microbial cell‐free DNA (mcfDNA) metagenomic sequencing test was sent on whole blood and detected *B. holmesii*. Given the clinicians’ uncertainty surrounding the significance of this pathogen, the patient remained off antimicrobials.

The patient was referred to our infectious diseases clinic a few weeks later. His physical examination at that point was remarkable for tenderness over the lumbar spinous processes. Repeat initial blood work revealed ESR 112 (2–38 mm/h) and CRP 118 (< 5.0 mg/L), with no leukocytosis, negative human immunodeficiency virus (HIV) screening, and negative bacterial, fungal, and mycobacterial blood cultures. Repeat plasma mcfDNA metagenomic sequencing test was sent on whole blood and again detected *B*. *holmesii*.

The infectious diseases team involved vascular surgery and orthopedic spine surgery teams, and the patient was admitted. He underwent aortic endograft explantation, debranching of celiac, superior mesenteric artery, bilateral renal arteries from descending aorta via left thoracotomy, repair of paravisceral aneurysm with aortobiiliac reconstruction with rifampin‐soaked Dacron graft, and debridement of L2‐L3 vertebral bodies and paraspinal tissues. Operative findings supported an extensive infectious process involving both the paraspinal region and the endograft. Neurosurgical debridement yielded purulent fluid and necrotic tissue involving the paraspinal tissues and anterior lumbar spine, while vascular exploration demonstrated liquefied thrombus and gross purulence surrounding the endograft, which was removed in one piece with both iliac limbs. Extensive debridement and abscess drainage were performed. No persistent endoleak was explicitly described in the operative documentation. Nine intraoperative samples from the aortic tissue, aortic graft, and spinal phlegmon and perispinal tissue were taken for microbiology studies, and the patient was started on empiric postoperative antimicrobial therapy with intravenous (IV) vancomycin with goal trough of 10–15 mcg/mL and cefepime 2 g IV every 12 h. He was later empirically switched to daptomycin 8 mg/kg IV daily and ertapenem 1 g IV daily for ease of outpatient administration pending discharge.

On Day 8 of incubation, *B. holmesii* grew on bacterial cultures from aortic tissue and was identified by matrix‐assisted laser desorption ionization–time‐of‐flight mass spectrometry (MALDI‐TOF MS). On postoperative Day 9, 16S rRNA gene sequencing from aortic tissue and spine tissue confirmed the identification. Unfortunately, the organism was not able to be grown at sufficient quantities to facilitate antimicrobial susceptibility testing, and following review of the literature pertaining to *B. holmesii* susceptibility patterns, his antimicrobial therapy was switched to meropenem IV 1 g every 8 h for a total of 12 weeks followed by oral trimethoprim/sulfamethoxazole (TMP/SMX) 800/160 mg orally (PO) twice daily for indefinite suppression due to vascular graft replacement.

**FIGURE 3:**
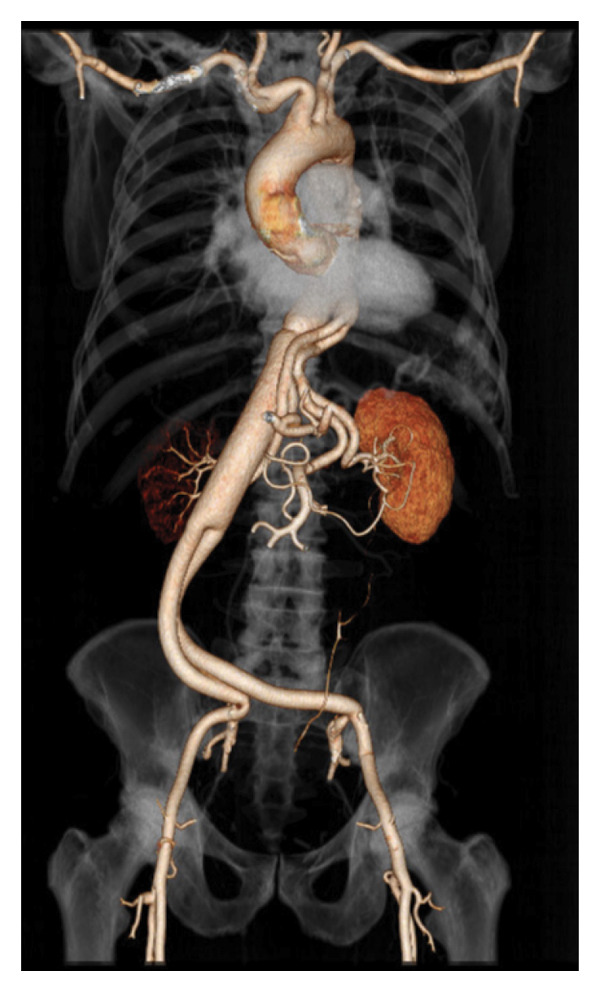
CT angiogram of abdomen and pelvis with angiographic reconstruction demonstrating infrarenal aortobiiliac endograft.

The patient remained under Infectious Diseases follow‐up every 3 months, reporting improvement in his lower back pain and normalization of his inflammatory markers. He experienced a drug‐related rash with systemic symptoms from TMP/SMX and was transitioned to doxycycline 100 mg PO twice daily and amoxicillin 500 mg PO twice daily with plans for TMP/SMX desensitization. A CT chest abdomen pelvis angiogram with IV contrast 3 months postoperatively did not identify an endoleak and demonstrated interval resection of the infected abdominal aortic endograft with a widely patent aortobiiliac bypass graft (Figure 3).

## 3. Discussion

*B. holmesii* is an infrequent human pathogen, predominantly reported in asplenic and other immunocompromised hosts [2, 3]. Two cases of spondylodiscitis have been reported in the literature, one in a patient with asplenia [4] and another one with intact immune system [5]. To date, however, no published case reports or case series have described VGIs attributed to *B. holmesii*.

The route of acquisition of *B. holmesii* in our patient remains unknown. No clear epidemiologic exposure or preceding respiratory illness was identified. Respiratory droplet transmission is considered the most likely source given the organism’s biological similarity to *B*. *pertussis* and its capacity to cause a comparable respiratory illness [6]. Respiratory and invasive *B. holmesii* isolates appear similar, suggesting that invasive disease may arise from secondary bloodstream spread after an initial respiratory infection [7]. This mechanism is biologically plausible in our case, particularly given the absence of an alternative source of bacteremia. Our patient may have developed bacteremia, with subsequent seeding of the vascular graft and spine by the pathogen.

Multiple approaches can be used to detect *B. holmesii* in clinical specimens and diagnose invasive infection [1]. Traditionally, the organism may be recovered by culture when suitable media are used [1]. However, although it is commonly used to diagnose invasive *B. holmesii* infection, it is often impractical and has limited sensitivity (12%–60%). In addition, specimens need to be incubated for at least 10 days to facilitate growth. Furthermore, the organism is inhibited by cephalexin, which is widely used in *Bordetella* culture media, and prompt transport to the laboratory [8]. These diagnostic limitations likely contribute to underrecognition of *B. holmesii* as a cause of invasive infection. These limitations may have contributed to the inability to identify the pathogen in the initial cultures from imaging‐guided biopsies. The subsequent biopsies were obtained surgically, providing higher‐quality specimens from both aortic tissue and the spine. In addition, results from the plasma mcfDNA metagenomic sequencing on whole blood and 16S rRNA gene sequencing of the tissue may have guided our microbiology laboratory to use appropriate culture media and extend incubation times. This may explain the subsequent positive culture results.

Identification can then be confirmed using PCR targeting specific genes of *B. holmesii*, 16S rRNA gene sequencing, or MALDI‐TOF MS. The nucleic acid–based techniques offer the advantage of remaining detectable later in the disease course and being less affected by prior or ongoing antibiotic therapy. In this context, there is no consensus or formal recommendation regarding the optimal diagnostic strategy to use to diagnose *B. holmesii*. In our reported case, *B. holmesii* was detected on two separate occasions by plasma mcfDNA metagenomic sequencing on whole blood despite prior negative results from conventional testing (cultures, broad‐range PCR). This test has been particularly helpful as an adjunctive test in diagnostically challenging cases, where it can contribute to organism identification and antimicrobial optimization when conventional diagnostics are unrevealing or delayed [9, 10]. Our case illustrated the potential utility of plasma mcfDNA sequencing in identifying fastidious organisms in deep‐seated infections when traditional microbiologic approaches are unable to identify the pathogen.

Our isolates had insufficient growth for susceptibility testing. Thus, a 12‐week course of meropenem was chosen based on a literature review and an extensive discussion with our Microbiology and Pharmacy teams, with close clinical and radiographic follow‐up to monitor treatment response. At present, no standardized treatment guidelines or antimicrobial susceptibility breakpoints exist for *B. holmesii* and further microbiological studies are needed to establish appropriate susceptibility testing breakpoints for clinical isolates. Resistance to cephalosporins, including ceftriaxone [1, 11] and cefotaxime [12], has been reported. Carbapenems and quinolones are the most reliable options. For example, a study from the Netherlands reported antimicrobial susceptibilities of 7 *B*. *holmesii* isolates and demonstrated median minimum inhibitory concentration (MIC) of 0.25 mg/L (range, 0.19–0.25 mg/L) for imipenem, 0.012 mg/L (0.012–0.16 mg/L) for meropenem, and 0.032 mg/L (0.032–0.125 mg/L) for ciprofloxacin [13]. In addition, a case of *B. holmesii* endocarditis was reported in which the blood isolate demonstrated MIC of 0.032 mg/L for both meropenem and moxifloxacin [12]. Another case of *B. holmesii* pericarditis was reported susceptibility to ciprofloxacin tested with an MIC < 0.12 μg/mL [14]. Similarly, 4 blood isolates were tested and demonstrated MIC ≤ 0.25 μg/mL for meropenem and ciprofloxacin [15]. A prolonged therapy, often exceeding 5–12 weeks, may be necessary, as shorter antimicrobial courses have been associated with relapse in some patients [1]. Management of this infection, therefore, relies heavily on extrapolation from limited case reports and expert opinions.

## 4. Conclusion

*B*. *holmesii* is an infrequent human pathogen, predominantly reported in asplenic and other immunocompromised hosts. To our knowledge, this is the first reported case of VGI caused by *B. holmesii* with concomitant NVO in an immunocompetent patient. This case highlights the value of advanced diagnostic tools, such as molecular testing and genomic sequencing, in identifying uncommon bacterial causes of invasive infectious diseases and guiding the diagnostic workup. To date, there are no standardized treatment guidelines or established antimicrobial susceptibility breakpoints for *B. holmesii*. As a result, the management of this infection largely relies on extrapolation from limited case reports and expert opinions.

## Funding

No funding was used for this project.

## Ethics Statement

The patient’s medical record was available for research use under Minnesota research authorization, and verbal consent was obtained from the patient.

## Conflicts of Interest

The authors declare no conflicts of interest.

## Data Availability

The data that support the findings of this study are available from the corresponding author upon reasonable request. The data are not publicly available due to privacy or ethical restrictions.

## References

[1] Pittet L. F. , Emonet S. , Schrenzel J. , Siegrist C.-A. , and Posfay-Barbe K. M. , *Bordetella holmesii*: An Under-recognised Bordetella Species, Lancet Infectious Diseases. (2014) 14, no. 6, 510–519, 10.1016/S1473-3099(14)70021-0, 2-s2.0-84901023266. 24721229

[2] Shepard C. W. , Daneshvar M. I. , Kaiser R. M. et al., *Bordetella holmesii* Bacteremia: A Newly Recognized Clinical Entity Among Asplenic Patients, Clinical Infectious Diseases: An Official Publication of the Infectious Diseases Society of America. (2004) 38, no. 6, 799–804, 10.1086/381888, 2-s2.0-12144285799. 14999621

[3] Tartof S. Y. , Gounder P. , Weiss D. et al., *Bordetella holmesii* Bacteremia Cases in the United States, April 2010-January 2011, Clinical Infectious Diseases: An Official Publication of the Infectious Diseases Society of America. (2014) 58, no. 2, e39–e43, 10.1093/cid/cit669, 2-s2.0-84891821541. 24092805 PMC4606926

[4] Fishbain J. T. , Riederer K. , Sawaf H. , and Mody R. , Invasive *Bordetella holmesii* Infections, Infectious Diseases (London, England). (2015) 47, no. 2, 65–68, 10.3109/00365548.2014.968609, 2-s2.0-84942606676. 25415654

[5] Nadji S. , Chopin M.-C. , Bourdon C. et al., Spondylodiscitis Caused by *Bordetella holmesii*, a Misrecognized Pathogen Emerging in Invasive Infections, International Journal of Infectious Diseases. (2018) 75, no. October, 95–97, 10.1016/j.ijid.2018.07.014, 2-s2.0-85053080345. 30031801

[6] Kamiya H. , Otsuka N. , Ando Y. et al., Transmission of *Bordetella holmesii* During Pertussis Outbreak, Japan, Emerging Infectious Diseases. (2012) 18, no. 7, 1166–1169, 10.3201/eid1807.120130, 2-s2.0-84862576197. 22709586 PMC3376812

[7] Pittet L. F. and Posfay-Barbe K. M. , *Bordetella holmesii*: Still Emerging and Elusive 20 Years on, Microbiology Spectrum. (2016) 4, no. 2, 10.1128/microbiolspec.EI10-0003-2015, 2-s2.0-85011272685. 27227292

[8] Mazengia E. , Silva E. A. , Peppe J. A. , Timperi R. , and George H. , Recovery of *Bordetella holmesii* From Patients With Pertussis-Like Symptoms: Use of Pulsed-Field Gel Electrophoresis to Characterize Circulating Strains, Journal of Clinical Microbiology. (2000) 38, no. 6, 2330–2333, 10.1128/JCM.38.6.2330-2333.2000. 10834997 PMC86794

[9] Huygens S. , Schauwvlieghe A. , Wlazlo N. et al., Diagnostic Value of Microbial Cell-Free DNA Sequencing for Suspected Invasive Fungal Infections: A Retrospective Multicenter Cohort Study, Open Forum Infectious Diseases. (2024) 11, no. 6, 10.1093/ofid/ofae252. PMC11166502 38868302

[10] Kim M. , Damronglerd P. , Molina Garcia S. et al., Illuminating the Challenges and Diagnostic Utility of Plasma Microbial Cell-Free DNA Sequencing in Suspected Infective Endocarditis: A Retrospective Observational Cohort Study, Open Forum Infectious Diseases. (2025) 12, no. 3, 10.1093/ofid/ofaf099. PMC11879537 40046887

[11] Monnier S. , Therby A. , Couzon B. , Doucet-Populaire F. , and Greder-Belan A. , Bactériémie à *Bordetella Holmesii* Chez Un Patient Drépanocytaire de 26 Ans, Medecine et Maladies Infectieuses. (2010) 40, no. 5, 299–301, 10.1016/j.medmal.2009.06.002, 2-s2.0-77952883965. 19586732

[12] Jonckheere S. , De Baere T. , Schroeyers P. , Soetens O. , De Bel A. , and Surmont I. , Prosthetic Valve Endocarditis Caused by *Bordetella holmesii*, an Acinetobacter Lookalike, Journal of Medical Microbiology. (2012) 61, no. 6, 874–877, 10.1099/jmm.0.038695-0, 2-s2.0-84861149112. 22403142

[13] Van Balen T. , Nieman An-E. , Hermans M. H. A. , Schneeberger P. M. , and de Vries E. , *Bordetella holmesii* Meningitis in a 12-Year-Old Anorectic Girl, The Pediatric Infectious Disease Journal. (2012) 31, no. 4, 421–422, 10.1097/INF.0b013e318241cd70, 2-s2.0-84858800637. 22315001

[14] Nei T. , Hyodo H. , Sonobe K. , Dan K. , and Saito R. , First Report of Infectious Pericarditis due to *Bordetella holmesii* in an Adult Patient With Malignant Lymphoma, Journal of Clinical Microbiology. (2012) 50, no. 5, 1815–1817, 10.1128/JCM.06772-11, 2-s2.0-84859997821. 22378902 PMC3347137

[15] Panagopoulos M. I. , Jean M. S. , Brun D. et al., *Bordetella holmesii* Bacteremia in Asplenic Children: Report of Four Cases Initially Misidentified as Acinetobacter lwoffii, Journal of Clinical Microbiology. (2010) 48, no. 10, 3762–3764, 10.1128/JCM.00595-10, 2-s2.0-77957799272. 20668129 PMC2953105

